# Peristaltic transfer of nanofluid with motile gyrotactic microorganisms with nonlinear thermic radiation

**DOI:** 10.1038/s41598-023-33995-2

**Published:** 2023-04-29

**Authors:** Doaa R. Mostapha, Nabil T. M. El-dabe

**Affiliations:** Department of Mathematics, Faculty of Education, Ain Shams University, Roxy, Cairo, Egypt

**Keywords:** Biotechnology, Physics

## Abstract

In situated theoretical article, a study of peristaltic transition of Jeffery nanofluid comprising motile gyrotactic microorganisms is exposed. The movement floods due to anisotropically stenosed endoscope influenced by Hall current, Joule heating during Darcy-Forchheimer feature. Influences of nonlinear thermic radiation, chemical interactions as well as Soret and Dufour scheme are exhibited. To ameliorate the competence of this article, activation energy has been appended to concentration of nano-particles due to the amended Arrhenius scheme and Buongiorno type. The slip stipulation is deemed relative to the speed scheme. Meanwhile, convective stipulation is reckoned for temperature. The proposition of protracted wavelength besides subdued Reynolds numeral is regulated to transit the manner of partial differential formulations that judges the fluid movement to ordinary one. Homotopy perturbation manner is tackled to manage the traditional solutions of generated neutralizations. Influences of assorted factors of the issue are debated and schematically showed with a class of charts. The situated study grants a medication for the malign cells and clogged arteries of the heart by manner of penetrating a slender tube (catheter). Also, this study may represent the depiction of the gastric juice movement in small intestine when an endoscope is permeating across it.

## Introduction

The nanofluid expression was first introduced by Choi^1^. Nanofluid is identified as a liquid involving nanoparticles. Nanoparticles have noticeable physicochemical, ocular, and organic features. Their tiny size allows them to enter the cell which exists in microscopic organisms, parasites and infections. Nanotechnology will promote remarkable advances in the detection and treatment of ailments. Over the past decade, nanofluids introduced fundamental implementations in improving the thermo-physical aspects, bacteriostatic action, nano-drug delivery, cancerous tissues, localized remedy and cancer remedy. These considerable implementations make various authors to investigate the action of nanofluids in varied positions, see Refs.^2–4^.

The advancement of non-Newtonian fluid**s** including nanoparticles seves a fundamental function in the human organ. Therefore, Jeffery liquid is deemed as one specific type of non-Newtonian fluids to describe the features of nanoparticles. Jeffrey model is regarded as the most significant non-Newtonian fluid type. Jeffrey type is a comparatively linear type in which the time derivatives are tackled in lieu of convected derivatives. It can be deemed as a blood model. Recently, sundry investigations have debated Jeffrey model through different states^5–7^.

In living organism, peristaltic movements are fundamentally formed by the shrinkage and elongation of some resilient organs. This grants a pressure force to convey fluids. It saves many biological implementations, for example, blood flows in veins, urine flows to bladder, and transmiton of medicines to coveted positions^8^. Further, there are some implementations in the biomedical tools like blood pumps and heart lung machines. The notion of the peristaltic transition through some suggestions, such as the approximation of protracted wavelength, feeble Reynolds numeral and trivial amplitude proportion was inspected^9,10^. Contemporary advancements on peristaltic system are tackled in some references as in Refs.^2,4–6^. The mechanism of peristaltic push of Jeffrey nanofluid in the presence of thermic radiation was investigated by Abbas et al.^7^. Further, Rashed and Ahmed^11^ debated the transition of peristalsis due to dusty Nanofluids over the curved channels.

Blood is pushed to body organs by a composite network of veins as well as arteries. Stenosis is regarded as an unusual straightening in the structure of blood vessel^12^. Also, It is defined by the expression “stricture”. Stenosis hinders the streaming of blood. In stenosed artery, the streaming of blood is stressed and resistance **is** higher than that of ordinary one^13^. Several authors have highlighted the study of stenosis. Rahman et al.^14^ debated the influences of Jeffrey nanoparticles in the presence of plain stenosis.

In the modern era, the nanofluid influx with thermic radiation, chemical interaction and activation energy have different uses in cooling, expulsion manner and polymer manufactureing. Chemical interactions can be categorized into two types of homogeneous as well as heterogeneous operations. The homogeneous type takes place permanently throughout elementary stages. By contrast, the heterogeneous one exists in a restricted phase. Thermic radiation and chemical interaction with activation energy are used in converting steam methane in porous media through solar-powered reactors Heat and mass transmission is counted as a paramount phenomenon in sundry engineering as well as industrial procedures, like, heat reciprocators, power collecting tools, nutrition processing and so on. Further, temperature gradients produce heat flux. It has many diverse uses in cooling processes, thermal regulation in electronic devices, and nuclear reactors^3,15^. Abbas et al.^7^ examined the effects of thermic radiation as well as chemical interaction due to propulsion of peristalsis of Jeffrey Nanofluid. The mechanism of peristaltic transition of couple stress liquid across the effect of chemical interaction as well as activation energy is examined^16^. Kumer et al.^17^ scrutinized the impact of nonlinear radiative heat and mass transfer on MHD mixed convection flow. Also, Kumer et al.^18^ checked the effect of nonlinear radiation of generalized Burgers nanofluid through a stretching sheet. Furthermore, Gireesha1 et al.^19^ discussed the numerical study of heat transfer for the aqueous suspensions of nanotubes with the effect of nonlinear thermic radiation. Kumer^20^ investigated the impact of heat transfer of nanofluid in the presence of slip factor.

Lately, analyzing strong magnetic field with low density is very important, which is distinguished as the Hall current. It has a wide rangr of applications in engineering areas like power generators and warming elements^21,22^. As well, Joule heating is generated while electricity force transmits to temperature via resistance^23–25^. Over and above, the influences of the Hall factor besides Joule heating on the peristaltic transition was examined^26^. Another important portion is porous media. Transfer across the porous construction is vital in the interaction the interaction among column wall and packing elements. Darcy’s procedure depends on tiny speed stipulations besides low porosity^27^. However, a new model proposes a modified formula by impeding the square idiom of speed in Darcy expression which is specified as the Forchheimer term^28^. The importance of Hall effects as well as Joule heating on the Darcy-Forchheimer peristaltic pushing was scrutinized^29^. Also, Kumer et al.^30^ checked the attributes of Joule heating and viscous dissipation of Oldroyd B nanofluid in the presence of thermic radiation. Further, Nazir et al.^31^ discussed the effect of Joule heating on thermal energy in ionized hyperbolic tangent object relied on hybrid nanomaterials.

Bioconvection is an influx produced by multilateral floating of motile microorganisms which are much less intensive than water. The self- pushed motile microorganisms condense the density of base fluids in a specific path. In the upper layer, the group of microorganisms leads to instability. Therefore, instability and producing of convection modalities happen. Moveable microorganisms are split into several kinds i.e. oxytactic, gyrotactic microorganisms, and negative gravitaxis^32,33^. The nanomaterials in the antimicrobial implementation enhance their antimicrobial influence. Advancement of non-Newtonian fluids including nanoparticles and microorganisms is an essential function in the human body. Nanoparticles are convenient for antibacterial encasement on catheters of blood vessels. The catheter is encasement with a mixture of anti-infection agents and nanoparticles to resist the structure of pivotal membranes. If the concentration of nanoparticle is little, bioconvection takes place in a nanofluid. In biological procedures, there has recently been considerable interest in flows involving nano-bioconvection^34^. Mechanisms of gyrotactic microorganisms in anti-infection agents throughout catheter or endoscope in the presence of nonlinear thermic radiation are examined^35^. Majeed et al.^36^ analysed the impact of thermic radiation in motile gyrotactic micro-organisms flow of nanoparticles. Further, Majeed et al.^37^ scrutinized the influence of radiative bioconvection flow in the presence of gyrotactic microorganism comprising activation energy. Also, Zhang et al.^38^ checked the impact of magnetic Reynolds numeral due to swimming of gyrotactic microorganisms among rotating plates. Bioconvection of nanofluid due to motile gyrotactic micro-organisms is investigated by many researchers, Refs.^39–43^.

In light of the aforementioned published works, the current paper investigates thermic radiation as well as the response of chemical interaction in a strong magnetized nanofluid influx. Investigation of the peristaltic transition of Jeffery fluid comprising motile gyrotactic microorganisms is carried out. The floods movement due to anisotropically stenosed endoscope is influenced by the Hall current, Joule heating and Darcy-Forchheimer feature. Influences of the Soret and Dufour scheme are exhibited. To promote the competence of this article, the activation energy has been implemented to concentration of nano-particles due to the amended Arrhenius scheme and Buongiorno type. The slip stipulation is deemed relative to the speed scheme. Meanwhile, convective stipulation is impleented in temperature. The proposition of protracted wavelength besides subdued Reynolds numeral is regulated to transform the partial differential formulations, which portray this phenomenon, to ordinary ones. Homotopy perturbation manner is used to manage the traditional solutions of generated neutralizations. The analytical solutions are exhibited in pictorial formula for speed, nanoparticle temperature, nanoparticle concentration, motile microorganisms and pressure gradient. Influences of the assorted factors of the issue are discussed and schematically showed with a set of charts. It is observed that the augmenting in local temperature Groshof numeral ${G}_{r}$ causes a boost in the velocity profile. Nevertheless, the rise in nanoparticle Groshof numeral ${R}_{N}$ attenuates velocity. Also, The Arrhenius expression is used to describe the mathematical formula of activation energy. Indeed, it is revealed that the drop in heat and acceleration are attributed to a weak reaction average constant. Furthermore, an elevation in Peclet numeral ${P}_{e}$ decreases the motile microorganism density, since robust Peclet numeral implies to stands for weaker Brownian coefficient which causes little penetration of swimming microorganism.

So, the motivation of this paper is to investigate peristaltic transport in the presence of mild stenosis which involves motile gyrotactic microorganisms in the presence of Hall current, Joule heating and Darcy-Forchheimer feature with the influences of the Soret and Dufour scheme. To promote the competence of this article, the activation energy has been implemented to concentration of nano-particles due to the amended Arrhenius scheme and Buongiorno type. The present study proposes a medication for the malign cells and clogged arteries of the heart by penetrating a slender tube (catheter). Also, it depicts the gastric juice movement in small intestine when an endoscope is permeating across it.

## Structure of the problem

**Figure 1:**
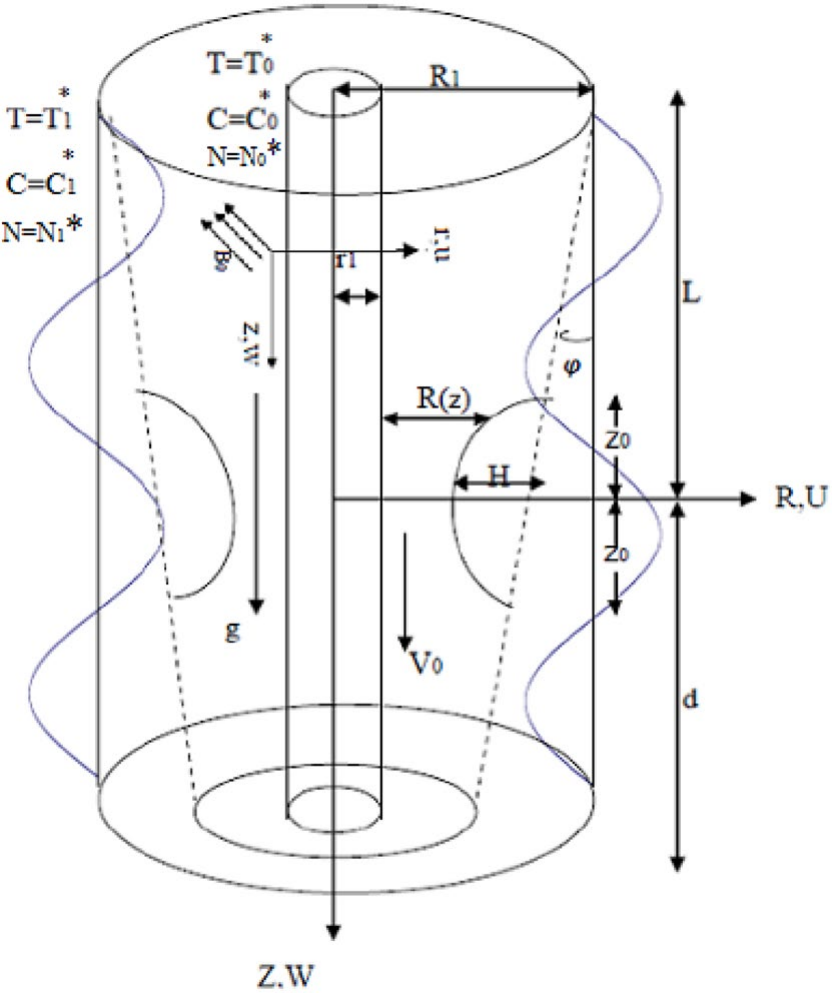
Description of natural prototype of the structure.

An unsteady locomotion of Jeffery nanofluid including motile gyrotactic microorganisms move through endoscope is exposed. There is a sinusoidal wave passing over the wall of outer anisotropically stenosed tube. In the interim, the inner tube is rigid, stable, and progressing with a stationary speed ${V}_{0}$. The influx proceeds down via a Darcy-Forchheimer porous surrounding. A stable sturdy magnetic field of strength ${B}_{0}$ acted upon the framework. The Hall and Joule heating actions are considered. The current paper addresses thermic radiation as well as the response of chemical interaction with Soret and Dufour scheme. Activation energy has been appended to concentration of nano-particles due to the amended Arrhenius scheme and Buongiorno type. Nevertheless, the electric field is overridden. For appropriateness, cylindrical co-ordinates ($R$, $\theta$, $Z$) are modulated. Further, the $Z$-axis exists along the tube axis. Permanence of the Hall current results in strength in the $\theta$- path. Thereafter, influx happens in three dimensions. For appropriateness, no impacts of temperature transmission, concentration and microorganisms characteristics are exposed in the path of $\theta$. Further, the slip stipulation is deemed relative to the speed scheme. Meanwhile, convective stipulation is implemented to temperature. Further, it is postulated that the inner tube at $r={r}_{1}$ is preserved at stationary temperature ${T}_{0}^{*}$, concentration ${C}_{0}^{*}$ and microorganism ${N}_{0}^{*}$. In addition, the outer tube is warmed by a temperature ${T}_{1}^{*}$, concentration ${C}_{1}^{*}$ and microorganism ${N}_{1}^{*}$, as shown in Fig. 1. This procedure brings about the heat transmission factor ${h}_{t}$. The magnetic Reynolds numeral is surmised to be extremely little quantity. Thence, the induced magnetic field becomes slight comparison with the external magnetic field. Thus, the induced magnetic field may be overridden. Delineation of the trouble is sketched in Fig. 1.

The radius $R(z)$ is modulated as^13^:

1 $$R(z)=\left\{\begin{array}{ll}{R}_{1}-\delta {m}_{1}(Z+L+d)& -{\mathrm{L}}^{*}\le \mathrm{Z}<-{\mathrm{Z}}_{0},\\ {R}_{1}-\delta {m}_{1}(Z+L+d)-\frac{{H}_{0}}{2}\left[1+\mathrm{cos}\frac{\pi Z}{{Z}_{0}}\right]& -{\mathrm{Z}}_{0}\le \mathrm{Z}\le {\mathrm{Z}}_{0},\\ {R}_{1}-\delta {m}_{1}(Z+L+d)& {\mathrm{Z}}_{0}<\mathrm{Z}\le \mathrm{d},\end{array}\right.$$

where, $$\delta =\frac{{R}_{1}}{\lambda }$$, $${H}_{0}=h\mathrm{cos}\phi$$ and $${m}_{1}=\mathrm{tan}\phi$$.

Proportion among ${H}_{0}$ and $r$ is revealed to be more slight than one. The outer tube is deemed to be restricted in length $L+d$^29^. This paper tackles all the conceivable distinct modulations of the outer tube. The converging state is specified at $\phi <0$, the non-tapered tube is assigned at $\phi =0$ as well as the diverging case is designated at $\phi >0$^44^.

The prototype non-Newtonian model is delineated by Jeffrey model. Consequently, the constitutive equation can be designated as^5^:

2 $$\underline{\underline{S}}=\frac{\mu }{1+{\lambda }_{1}}\left(\underline{\underline{A}}+{\lambda }_{2}\underline{\underline{A}}\right),$$

where (dot) implies to the differentiation with respect to time,

3 $$\underline{\underline{A}}=\nabla \underline{V}+\nabla {\underline{V}}^{T},$$

With regard to the incompressibility feature, the continuity equation is converted as:

4 $$\nabla .\underline{V}=0.$$

The controlling equation of movement is constructed as^23,26^:

5 $${\rho }_{f}\left(\frac{\partial \underline{V}}{\partial t}+(\underline{V}.\nabla )\underline{V}\right)=-\nabla P+\nabla .\underline{\underline{S}}+\underline{J}\wedge \underline{B}-\frac{\mu }{{k}_{1}}\underline{V}-\frac{{c}_{b}}{\sqrt{{k}_{1}}}\underline{V}|\underline{V}|+{\rho }_{f}\underline{g},$$

where $\underline{g}=(\mathrm{0,0},-g)$, $\underline{J}\wedge \underline{B}$ signifies the Lorentz force, and $\underline{B}=(0,{B}_{0},0)$. The current density is detected due to the concept of overriding polarization voltage ($\underline{E}=0$)^21^. Thenceforward, one gets:

6 $$\underline{J}=\sigma \left(\underline{V}\wedge \underline{B}-\frac{1}{e{n}_{c}}\underline{J}\wedge \underline{B}\right).$$

The density of nanofluids is formulated as^28^

7 $$\rho ={\rho }_{f}{T}_{e}(1-{C}_{1}^{*})(T-{T}_{1}^{*})-({\rho }_{p}-{\rho }_{f}){C}_{e}(C-{C}_{1}^{*})-(N-{N}_{1}^{*})\vartheta ({\rho }_{m}-{\rho }_{f}),$$

The equation of energy in the presence of nonlinear thermic radiation and Dufour effects may be designated as^27^:

8 $$(\rho c{)}_{f}\left(\frac{\partial T}{\partial t}+\underline{V}.\nabla T\right)=K{\nabla }^{2}T-\nabla .{q}_{r}+\frac{1}{\sigma }\underline{J}.\underline{J}+\frac{\mu }{{k}_{1}}\left(U+W\right)\left|\underline{V}\right| +(\rho c{)}_{p}\left({D}_{B}\nabla C.\nabla T+\frac{{D}_{T}}{{T}_{1}^{*}}\nabla T.\nabla T\right)+\frac{D{K}_{T}}{{C}_{s}}{\nabla }^{2}C,$$

where $$k=\frac{K}{(\rho c{)}_{f}}$$ and $$|\underline{V}|=\sqrt{{U}^{2}+{W}^{2}}$$.

Complying the Rosseland diffusion flux model^35^, the nonlinear radiative heat flux may be designated as:

9 $${q}_{r}=-\frac{4{\sigma }_{0}}{3{K}_{0}}\frac{\partial {T}^{4}}{\partial R},$$

The differences in temperature are regarded to be weak. Thus, ${T}^{4}$ indicates a linear function of temperature. Tracing Talyor series about mean temperature ${T}_{m}$ and overriding the higher orders, the nonlinear radiative temperature is switched to:

10 $${q}_{r}=-\frac{16{\sigma }_{0}}{3{K}_{0}}{T}_{m}\frac{\partial T}{\partial R}.$$

Equation of concentration in the attendance of chemical interaction as well as activation energy^4^ is designated as:

11 $$\frac{\partial C}{\partial t}+\underline{V}.\nabla C={D}_{B}{\nabla }^{2}C+\frac{{D}_{T}{K}_{T}}{{T}_{m}}{\nabla }^{2}T-{k}_{r}^{2}\left(C-{C}_{1}^{*}\right)\left(\frac{T}{{T}_{1}^{*}}\right)^{n}\mathrm{exp}\left(\frac{-{E}_{a}}{\omega T}\right),$$

where ($-1<n<1$). The latest term in Eq. (11) is realized as "Arrhenius term". Arrhenius term can describe the impacts of chemical interaction as well as activation energy commingled with a nanofluid.

The equation of motile microorganism is formulated as^35^:

12 $$\frac{\partial N}{\partial t}+\underline{V}.\nabla N+\frac{b{\omega }_{e}}{({C}_{0}^{*}-{C}_{1}^{*})}\nabla .(N\nabla C)={D}_{m}{\nabla }^{2}N,$$

The peristaltic wall surface may be designated as^10^:

13 $${H}_{1}={a}_{0}\mathrm{cos}\frac{2\pi }{\lambda }\left(Z-\frac{kt}{{R}_{1}}\right).$$

Boundary stipulations can be expressed as

$$U=\frac{\partial {H}_{1}}{\partial t}, W=-\gamma {S}_{RZ}, K\frac{\partial T}{\partial r}=-{h}_{t}(T-{T}_{1}^{*}),$$

14 $$C={C}_{1}^{*}, N={N}_{1}^{*} at R={R}_{2}=R(z)+{H}_{1},$$

15 $$U=0, w={V}_{0}, T={T}_{0}^{*}, C={C}_{0}^{*}, N={N}_{0}^{*} at R={r}_{1},$$

where ${h}_{t}$ implies to the proportionality constant among the flux of temperature and the thermodynamic driving force.

In the fixed frame, the volume flow rate is recognized as^9^:

16 $$Q=2\pi {\int }_{{r}_{1}}^{{R}_{2}}WRdR,$$

$${R}_{2}={R}_{2}\left(Z,t\right).$$

At a fixed Z-posture, time averaged $\widehat{Q}$ over duration $\tau =\frac{\lambda {R}_{1}}{k}$ is reported as

17 $$\widehat{Q}=\frac{1}{\tau }{\int }_{0}^{\tau }Qd\tau .$$

The variation in the pressure at the ends of the tube is regarded to be steady. Thus, in the laboratory coordinate $(R,0,Z)$, the unsteady inflow renders into steady steady one which happens in the wave coordinate $(r,0,z)$. The transmission among the two coordinates may be designated as

18 $$u=U, w=W-\frac{k}{{R}_{1}}, r=R, \mathrm{and}\, z=Z-\frac{k}{{R}_{1}}t.$$

The proposition of protracted wavelength besides subdued Reynolds numeral is regulated to transit the manner of partial differential formulations that judges the fluid movement to ordinary one^9^. It is adequate to reformulate the former equations in compatible dimensionless formulations. This can be conducted by a mechanism counting preponderantly on the merits of length and mass. The next non-dimensional idioms based on length ${R}_{1}$ and $\lambda$, besides the mass $M$. The other non-dimensional amounts are as follows

19 $$\begin{aligned} & \overline{r}=\frac{r}{{R}_{1}}, \overline{z}=\frac{z}{\lambda }, \overline{u}=\frac{u{R}_{1}}{k\delta }, \overline{w}=\frac{w{R}_{1}}{k}, \overline{{H}_{1}}=\frac{{H}_{1}}{{R}_{1}}, \delta =\frac{{R}_{1}}{\lambda }, \overline{S}=\frac{S{R}_{1}^{2}}{\mu k}, \overline{{z}_{0}}=\frac{{z}_{0}}{\lambda },\\ & \overline{L}=\frac{L}{\lambda }, \overline{h}=\frac{h}{{R}_{1}}, \overline{d}=\frac{d}{\lambda }, \overline{R\left(z\right)}=\frac{R\left(z\right)}{{R}_{1}}, \overline{P}=\frac{P{R}_{1}^{3}}{\mu \lambda k}, \overline{T}=\frac{T-{T}_{1}^{*}}{\beta {R}_{1}}, \overline{C}=\frac{C-{C}_{1}^{*}}{{C}_{0}^{*}-{C}_{1}^{*}},\\ & \overline{N}=\frac{N-{N}_{1}^{*}}{{N}_{0}^{*}-{N}_{1}^{*}} \mathrm{and} \overline{Q}=\frac{Q}{2\pi {R}_{1}k}.\end{aligned}$$

Regarding the other dimensionless numerals are as follows: ${\lambda }^{*}=\frac{{\lambda }_{2}k}{{R}_{1}^{2}}$, ${R}_{N}=\frac{{R}_{1}^{3}{C}_{e}({C}_{0}^{*}-{C}_{1}^{*})g({\rho }_{p}-{\rho }_{f})}{\mu k}$, ${G}_{r}=\frac{{\rho }_{f}{T}_{e}(1-{C}_{1}^{*})(\beta {R}_{1}){R}_{1}^{3}g}{\mu k}$, ${R}_{b}=\frac{{R}_{1}^{3}g({N}_{0}^{*}-{N}_{1}^{*})({\rho }_{m}-{\rho }_{f})\vartheta }{\mu k}$, ${D}_{a}=\frac{{k}_{1}}{{R}_{1}^{2}}$, ${F}_{r}=\frac{{c}_{b}{R}_{1}k}{\nu \sqrt{{k}_{1}}}$, ${R}_{e}=\frac{k}{\nu }$, $\nu =\frac{\mu }{\rho }$, $\epsilon ={a}_{0}/{R}_{1}$, ${H}^{2}=\frac{\sigma {B}_{0}^{2}{R}_{1}^{2}}{\mu }$, $m=\frac{\sigma {B}_{0}}{e{n}_{c}}$, ${P}_{r}=\frac{\mu }{\rho k}$, ${\tau }_{c}=\frac{(\rho c{)}_{p}}{(\rho c{)}_{f}}$, ${N}_{b}=\frac{{\tau }_{c}{D}_{B}({C}_{0}^{*}-{C}_{1}^{*})}{k}$, ${N}_{t}=\frac{{\tau }_{c}{D}_{T}(\beta {R}_{1})}{k{T}_{1}^{*}}$, ${E}_{c}=\frac{{k}^{2}}{{R}_{1}^{3}{c}_{p}\beta }$, ${B}_{r}={P}_{r}{E}_{c}$, ${R}_{n}=\frac{16{\sigma }_{0}{T}_{m}^{3}}{3K{K}_{0}}$, ${D}_{u}=\frac{D{K}_{T}({C}_{0}^{*}-{C}_{1}^{*})}{\mu {C}_{s}\beta {R}_{1}{c}_{f}}$, ${L}_{e}=\frac{k}{{D}_{B}}$, ${S}_{c}=\frac{k}{D}$, $\alpha =\frac{{k}_{r}^{2}{R}_{1}^{2}}{k}$, ${\beta }_{t}=\frac{\beta {R}_{1}}{{T}_{1}^{*}}$, $\xi =\frac{{E}_{a}}{\omega {T}_{1}^{*}}$, ${L}_{m}=\frac{k}{{D}_{m}}$, ${P}_{e}=\frac{b{\omega }_{e}}{{D}_{m}}$, $\Omega =\frac{{N}_{1}^{*}}{{N}_{0}^{*}-{N}_{1}^{*}}$, ${\gamma }^{*}=-\frac{\gamma \mu }{{R}_{1}}$ and ${\beta }_{h}=\frac{{h}_{t}{R}_{1}}{k}$. The bars sign assigns the non-dimensional amounts. Henceforth, these will be disregarded for simplicity.

The radial speed $u$ is frequently insignificant in proximity with the pivotal one $w$^13^. In addendum, alteration in the $z$-trend is weaker than that in the radial one. Hence, hypothize that $u<<w$ and $\frac{\partial w}{\partial z}<<\frac{\partial w}{\partial r}$ . Moreover, the consideration of the expanded wave length approach $\delta <<1$ is regarded. Presently, $\delta$ is extremely prosaic. Suitably, we display an approximate solution for Eqs. (2), (3), (4), (5), (6), (7), (8), (9), (10), (11), (12), (13), (14) and (15) by inspecting a perturbation mechanism in the weak factor $\delta$. This mechanism may be designated as:

The momentum equation in the $r$-trend converts into:

20 $$\frac{\partial P}{\partial r}=0,$$

where Eq. (20) clarifies that $P$ is in $z$ only.

The $z$-component of the momentum equation turns to:

21 $$\begin{aligned}\frac{\partial P}{\partial z}&=\frac{1}{r(1+{\lambda }_{1})}\frac{\partial }{\partial r}\left(r\frac{\partial w}{\partial r}\right)+\frac{{\lambda }^{*}\delta }{r(1+{\lambda }_{1})}\frac{\partial }{\partial r}\left(rw\frac{{\partial }^{2}w}{\partial r\partial z}\right)-\left(\frac{{H}^{2}}{1+{m}^{2}}+\frac{1}{{D}_{a}}\right)(w+1)\\ &\quad-{F}_{r}(w+1{)}^{2}+\delta {R}_{e}w\frac{\partial w}{\partial z}+{G}_{r}T-{R}_{N}C-{R}_{b}N.\end{aligned}$$

The heat equation outcomes:

$$(1+{R}_{n})\frac{1}{r}\frac{\partial }{\partial r}(r\frac{\partial T}{\partial r})+\frac{{D}_{u}{P}_{r}}{1+{R}_{n}}\frac{1}{r}\frac{\partial }{\partial r}(r\frac{\partial C}{\partial r})+{B}_{r}\left(\frac{{H}^{2}}{{m}^{2}+1}+\frac{1}{{D}_{a}}\right)(w+1{)}^{2}$$

22 $$+{N}_{b}\frac{\partial C}{\partial r}\frac{\partial T}{\partial r}+{N}_{t}(\frac{\partial T}{\partial r}{)}^{2}=\delta w\frac{\partial T}{\partial z}.$$

The concentration equation provides:

23 $$\frac{1}{r}\frac{\partial }{\partial r}\left(r\frac{\partial C}{\partial r}\right)+\frac{{N}_{t}}{{N}_{b}r}\frac{\partial }{\partial r}\left(r\frac{\partial T}{\partial r}\right)-\alpha {S}_{c}{\mathrm{exp}}^{-\xi }(1+n{\beta }_{t}T)(1-\xi {\beta }_{t}T)={L}_{e}\delta w\frac{\partial C}{\partial z}.$$

Equation of motile microorganism is formulated as

24 $$\frac{1}{r}\frac{\partial }{\partial r}\left(r\frac{\partial N}{\partial r}\right)-{P}_{e}\left((N+\Omega )\frac{{\partial }^{2}C}{\partial {r}^{2}}+\frac{\partial N}{\partial r}\frac{\partial C}{\partial r}\right)={L}_{m}\delta w\frac{\partial N}{\partial z}.$$

The dimensionless volume flow average in the movable coordinate mechanism is converted to:

25 $$q={\int }_{{r}_{1}}^{{r}_{2}}wrdr.$$

The proper dimensionless boundary stipulations become:

$$u=2\pi \epsilon\, \mathrm{sin}2\pi \,z, \,w=-1-\frac{{\gamma }^{*}}{1+{\lambda }_{1}}\left(\frac{\partial w}{\partial r}-2{\lambda }^{*}\delta w\frac{{\partial }^{2}w}{\partial r\partial z}\right), \frac{\partial T}{\partial r}=-{\beta }_{h}T,$$

26 $$C=0,\, N=0\, at\, r={r}_{2}=r(z)+{H}_{1},$$

27 $$u=0,\, w={V}_{0}^{*},\, T=1,\, C=1,\, N=1\, at\, r={r}_{1},$$

where

28 $${H}_{1}=\epsilon\, \mathrm{cos}2\pi z.$$

The dimensionless $R(z)$ may be expressed as:

29 $$R(z)=\left\{\begin{array}{ll}1-{m}_{1}(z+L+d)& -{\rm{L}} \le {\rm{z}} < -{\rm{z}}_{0},\\ 1-{m}_{1}(z+L+d)-\frac{h{\rm{cos}}\phi }{2}[1+{\rm{cos}} \frac{\pi z}{{L}_{0}}]& -{\rm{z}}_{0}\le {\rm{z}} \le {\rm{z}}_{0},\\ 1-{m}_{1}(z+L+d)&{\rm{z}}_{0} < {\rm{z}} \le {\rm{d}}.\end{array}\right.$$

The posterior Section tackles the traditional solutions of this mechanism based on a perturbation principal in the prosaic factor $\delta$ as well as HPM.

## Mechanism of solution

To tackle the nonlinear mechanism which is expressed by Eqs. (21), (22), (23), (24) and (25) along the suitable boundary conditions (26) as well as (27), it is supposed that any structure, such as $w$, $P$, $T$, $C$ and $N$ may be formulated as:

30 $$\zeta ={\zeta }_{0}+\delta {\zeta }_{1}+........,$$

where ${\zeta }_{0}$ stands for zero order of $\delta$ and the first one is assigned via ${\zeta }_{1}$.subrogating Eq. (30) into the mechanism of Eqs. (21), (22), (23), (24) and (25) and grouping terms of similar powers of $\delta$, which produces zero as well as first order mechanisms of partial differential equations with the suitable boundary stipulations. In the following subsections, we will address these orders:

### Zero-order scheme in term of $\delta$

Due to zero-order steps, the fluid turns to a Newtonian fluid (in disappearing of $\delta$). Hence, controlling equations of flow will appear with overriding the non-Newtonian coefficient. Thence, the momentum equation in $z$-direction converts into:

31 $$\frac{d{P}_{0}}{dz}=\frac{1}{r(1+{\lambda }_{1})}\frac{\partial }{\partial r}\left(r\frac{\partial {w}_{0}}{\partial r}\right)-\left(\frac{{H}^{2}}{1+{m}^{2}}+\frac{1}{{D}_{a}}\right)({w}_{0}+1)-{F}_{r}({w}_{0}+1{)}^{2}+{G}_{r}T-{R}_{N}C-{R}_{b}N,$$

the heat equation provides:

32 $$(1+{R}_{n})\frac{1}{r}\frac{\partial }{\partial r}\left(r\frac{\partial {T}_{0}}{\partial r}\right)+\frac{{D}_{u}{P}_{r}}{1+{R}_{n}}\frac{1}{r}\frac{\partial }{\partial r}\left(r\frac{\partial {C}_{0}}{\partial r}\right)+{B}_{r}\left(\frac{{H}^{2}}{{m}^{2}+1}+\frac{1}{{D}_{a}}\right)({w}_{0}+1{)}^{2} +{N}_{b}\frac{\partial {C}_{0}}{\partial r}\frac{\partial {T}_{0}}{\partial r}+{N}_{t}\left(\frac{\partial {T}_{0}}{\partial r}\right)^{2}=0,$$

the concentration equation yeilds:

33 $$\frac{1}{r}\frac{\partial }{\partial r}\left(r\frac{\partial {C}_{0}}{\partial r}\right)+\frac{{N}_{t}}{{N}_{b}r}\frac{\partial }{\partial r}\left(r\frac{\partial {T}_{0}}{\partial r}\right)-\alpha {S}_{c}{\mathrm{exp}}^{-\xi }(1+n{\beta }_{t}{T}_{0})(1-\xi {\beta }_{t}{T}_{0})=0,$$

equation of motile microorganism turns to:

34 $$\frac{1}{r}\frac{\partial }{\partial r}\left(r\frac{\partial {N}_{0}}{\partial r}\right)-{P}_{e}\left((N+\Omega )\frac{{\partial }^{2}{C}_{0}}{\partial {r}^{2}}+\frac{\partial {N}_{0}}{\partial r}\frac{\partial {C}_{0}}{\partial r}\right)=0,$$

and the adequate boundary stipulations may be expressed as

$${u}_{0}=2\pi \epsilon \mathrm{sin}2\pi z, {w}_{0}=-1-\frac{{\gamma }^{*}}{1+{\lambda }_{1}}\frac{\partial {w}_{0}}{\partial r}, \frac{\partial {T}_{0}}{\partial r}=-{\beta }_{h}{T}_{0},$$

35 $${C}_{0}={N}_{0}=0 \; at \; r={r}_{2}=r(z)+{H}_{1},$$

36 $${u}_{0}=0, {w}_{0}={V}_{0}^{*}, {T}_{0}={C}_{0}={N}_{0}=1 \; at \; r={r}_{1}.$$

The conjunction among the HPM and the traditional perturbation mechanism revealed that the expression is more robust and efficacious. This mechanism overrides obstacles that stick on the classical perturbation mechanism. The HPM does not claim any little parameter in the introduced equation^45^. HPM regards a little factor ${p}_{h}\in [\mathrm{0,1}]$. Hence, HPM is implemented to non-linear differential Eqs. (31), (32), (33) and (34) with the suitable boundary conditions (35) and (36) as follows^45^:

37 $$\begin{aligned}H({w}_{0},{p}_{h})&=LI({w}_{0})-LI({w}_{00})+{p}_{h}LI({w}_{00})+{p}_{h}[\frac{1}{r(1+{\lambda }_{1})}\frac{\partial }{\partial r}\left(r\frac{\partial {w}_{0}}{\partial r}\right)\\ &\quad-\left(\frac{{H}^{2}}{1+{m}^{2}}+\frac{1}{{D}_{a}}\right)({w}_{0}+1)-{F}_{r}({w}_{0}+1{)}^{2}+{G}_{r}T-{R}_{N}C-{R}_{b}N],\end{aligned}$$

38 $$\begin{aligned}H({T}_{0},{p}_{h})&=LI({T}_{0})-LI({T}_{00})+{p}_{h}LI({T}_{00})+{p}_{h}\left[(1+{R}_{n})\frac{1}{r}\frac{\partial }{\partial r}\left(r\frac{\partial {T}_{0}}{\partial r}\right)+\frac{{D}_{u}{P}_{r}}{1+{R}_{n}}\frac{1}{r}\frac{\partial }{\partial r}\left(r\frac{\partial {C}_{0}}{\partial r}\right)\right. \\&\quad\left.+{B}_{r}\left(\frac{{H}^{2}}{{m}^{2}+1}+\frac{1}{{D}_{a}}\right)({w}_{0}+1{)}^{2}+{N}_{b}\frac{\partial {C}_{0}}{\partial r}\frac{\partial {T}_{0}}{\partial r}+{N}_{t}\left(\frac{\partial {T}_{0}}{\partial r}\right)^{2}\right],\end{aligned}$$

39 $$H({C}_{0},{p}_{h})=LI({C}_{0})-LI({C}_{00})+{p}_{h}LI({C}_{00})++{p}_{h}\left[\frac{1}{r}\frac{\partial }{\partial r}(r\frac{\partial {C}_{0}}{\partial r})+\frac{{N}_{t}}{{N}_{b}r}\frac{\partial }{\partial r}(r\frac{\partial {T}_{0}}{\partial r})-\alpha {S}_{c}{\mathrm{exp}}^{-\xi }(1+n{\beta }_{t}{T}_{0})(1-\xi {\beta }_{t}{T}_{0})\right],$$

40 $$H({N}_{0},{p}_{h})=LI({N}_{0})-LI({N}_{00})+{p}_{h}LI({N}_{00})++{p}_{h}\left[\frac{1}{r}\frac{\partial }{\partial r}\left(r\frac{\partial {N}_{0}}{\partial r}\right)-{P}_{e}\left((N+\Omega )\frac{{\partial }^{2}{C}_{0}}{\partial {r}^{2}}+\frac{\partial {N}_{0}}{\partial r}\frac{\partial {C}_{0}}{\partial r}\right)\right],$$

To get the premier surmise for the speed ${w}_{0}$, the linear factor $LI$ may be regarded as $LI=\frac{1}{r}\frac{\partial }{\partial r}\left(r\frac{\partial }{\partial r}\right)-\frac{d{P}_{0}}{dz}$. Accordingly, the initial surmize ${w}_{00}$ is formulated as:

41 $${w}_{00}\left(r,z\right)=\left(1+{\lambda }_{1}\right){G}_{0}\left(z\right)\frac{{r}^{2}}{4}+{d}_{1}\mathrm{ln}r+{d}_{2},$$

$${G}_{0}(z)=\frac{d{P}_{0}}{dz}$$

The solution ${w}_{00}(r,z)$ interprets the speed profile as far as original influx. The original influx signifies local Hagen-Poiseuille before peristalsis tacks place.

The linear factor $LI=\frac{1}{r}\frac{\partial }{\partial r}\left(r\frac{\partial }{\partial r}\right)$ is designated to expose the premier surmise for temperature ${T}_{00}$, concentration ${C}_{00}$ and microorganism ${N}_{00}$.

42 $${T}_{00}(r,z)=\frac{\mathrm{ln}r+{b}_{1}}{{b}_{2}}.$$

43 $${C}_{00}(r,z)=\frac{\mathrm{ln}r-\mathrm{ln}{r}_{2}(z)}{\mathrm{ln}{r}_{1}-\mathrm{ln}{r}_{2}(z)},$$

44 $${N}_{00}(r,z)=\frac{\mathrm{ln}r-\mathrm{ln}{r}_{2}(z)}{\mathrm{ln}{r}_{1}-\mathrm{ln}{r}_{2}(z)},$$

The intrinsic presumption is that the profiles ${w}_{0}, {T}_{0}, {C}_{0}$ and ${N}_{0}$ can be broadened as:

45 $${\varsigma }_{0}={\varsigma }_{00}+{p}_{h}{\varsigma }_{11}+...,$$

where ${\varsigma }_{00}$ stands for zero order of ${p}_{h}$ and the first one is assigned via ${\varsigma }_{11}$.

The solutions of pivotal speed, temperature, concentration and microorganism in state of ${p}_{h}=1$ may be expressed as:

46 $${w}_{0}\left(r,z\right)=\frac{{a}_{5}}{36}{r}^{6}+{a}_{11}{r}^{4}+\frac{{a}_{8}}{16}{r}^{4}\mathrm{ln}r+\left(\frac{\left(1+{\lambda }_{1}\right){G}_{0}\left(z\right)}{4}+{a}_{12}\right){r}^{2}+{a}_{13}{r}^{2}\mathrm{ln}r+\frac{{a}_{7}}{4}{r}^{2}{\mathrm{ln}}^{2}r+({d}_{3}+{d}_{1})\mathrm{ln}r+{d}_{4}+{d}_{2},$$

47 $${T}_{0}(r,z)=\frac{{a}_{17}}{6}{r}^{6}+{a}_{24}{r}^{4}+\frac{{a}_{20}}{4}{r}^{4}\mathrm{ln}r+{a}_{25}{r}^{2}+{a}_{26}{r}^{2}\mathrm{ln}r+\frac{{a}_{22}}{2}{r}^{2}{\mathrm{ln}}^{2}r+\frac{{a}_{23}}{2}{\mathrm{ln}}^{2}r+({d}_{5}+\frac{1}{{b}_{2}})\mathrm{ln}r+{d}_{6}+\frac{{b}_{1}}{{b}_{2}},$$

48 $${C}_{0}(r,z)={a}_{30}{r}^{2}+{a}_{31}{r}^{2}\mathrm{ln}r+\frac{{a}_{29}}{2}{r}^{2}{\mathrm{ln}}^{2}r+\left({d}_{7}+\frac{1}{\mathrm{ln}{r}_{1}-\mathrm{ln}{r}_{2}(z)}\right)\mathrm{ln}r+{d}_{8} -\frac{\mathrm{ln}{r}_{2}(z)}{\mathrm{ln}{r}_{1}-\mathrm{ln}{r}_{2}(z)},$$

49 $${N}_{0}(r,z)={P}_{e}\left(-\frac{{\mathrm{ln}}^{3}r}{6(\mathrm{ln}{r}_{1}-\mathrm{ln}{r}_{2}(z){)}^{2}}+\frac{{a}_{32}}{2}{\mathrm{ln}}^{2}r\right)+({d}_{9}+\frac{1}{\mathrm{ln}{r}_{1}-\mathrm{ln}{r}_{2}(z)})\mathrm{ln}r+{d}_{10}-\frac{\mathrm{ln}{r}_{2}(z)}{\mathrm{ln}{r}_{1}-\mathrm{ln}{r}_{2}(z)},$$

Currently, the volume flow rate is used to estimate ${G}_{0}(z)$.

By merging Eqs. (17) and (25):

50 $${Q}_{0}={q}_{0}+({r}_{2}(z{)}^{2}-{r}_{1}^{2}),$$

from Eq. (18)

51 $${\widehat{Q}}_{0}={q}_{0}+\frac{1}{4}\left(2({\mathrm{R}}^{2}(\mathrm{z})-{\mathrm{r}}_{1}^{2})+{\epsilon }^{2}\right).$$

Hence, the solution of ${G}_{0}(z)$ can be designated as

52 $${G}_{0}(z)=\frac{16\left({\widehat{Q}}_{0}-\frac{1}{2}({R}^{2}-{r}_{1}^{2})-\frac{{\epsilon }^{2}}{4}+\frac{1}{4}({a}_{2}-2{a}_{4})({r}_{2}^{2}-{r}_{1}^{2})-\frac{{a}_{2}}{2}({r}_{2}^{2}\mathrm{ln}{r}_{2}-{r}_{1}^{2}\mathrm{ln}{r}_{1})\right)}{(1+{\lambda }_{1})({r}_{2}^{4}-{r}_{1}^{4})+4(2{a}_{3}-{a}_{1})({r}_{2}^{2}-{r}_{1}^{2})+8{a}_{1}({r}_{2}^{2}\mathrm{ln}{r}_{2}-{r}_{1}^{2}\mathrm{ln}{r}_{1})},$$

where the constants ${d}_{1},$ ${d}_{2},$…..${,d}_{10}$ and ${a}_{1},{a}_{2}{,\dots .., a}_{32}$ are determined but they are not involved in this article to reduce the length.

### First-order mechanism in term of $\delta$

The former zero-order mechanism produced in the last subsection will be inserted in the first-order controlled mechanism to carry out the non-homogeneous combination of linear partial differential equations. This mechanism will be expressed as:

The momentum equation in the $z$-directions converts into:

53 $$\begin{aligned}\frac{\partial {P}_{1}}{\partial z}&=\frac{1}{r(1+{\lambda }_{1})}\frac{\partial }{\partial r}\left(r\frac{\partial {w}_{1}}{\partial r}\right)+\frac{{\lambda }^{*}}{r(1+{\lambda }_{1})}\frac{\partial }{\partial r}\left(r\frac{\partial {w}_{0}}{\partial r}\frac{{\partial }^{2}{w}_{0}}{\partial r\partial z}\right)-\left(\frac{{H}^{2}}{1+{m}^{2}}+\frac{1}{{D}_{a}}\right){w}_{1}\\ &\quad-2{F}_{r}({w}_{0}+1){w}_{1}+{R}_{e}{w}_{0}\frac{\partial {w}_{0}}{\partial z}+{G}_{r}{T}_{1}-{R}_{N}{C}_{1}-{R}_{b}{N}_{1}.\end{aligned}$$

The heat equation yields:

54 $$\begin{aligned} & (1+{R}_{n})\frac{1}{r}\frac{\partial }{\partial r}\left(r\frac{\partial {T}_{1}}{\partial r}\right)+\frac{{D}_{u}{P}_{r}}{1+{R}_{n}}\frac{1}{r}\frac{\partial }{\partial r}\left(r\frac{\partial {C}_{1}}{\partial r}\right)+2{B}_{r}\left(\frac{{H}^{2}}{{m}^{2}+1}+\frac{1}{{D}_{a}}\right)({w}_{0}+1){w}_{1}\\ &\quad+{N}_{b}\left(\frac{\partial {C}_{0}}{\partial r}\frac{\partial {T}_{1}}{\partial r}+\frac{\partial {C}_{1}}{\partial r}\frac{\partial {T}_{0}}{\partial r}\right)+2{N}_{t}\frac{\partial {T}_{0}}{\partial r}\frac{\partial {T}_{1}}{\partial r}={w}_{0}\frac{\partial {T}_{0}}{\partial z}.\end{aligned}$$

The concentration equation provides:

55 $$\frac{1}{r}\frac{\partial }{\partial r}\left(r\frac{\partial {C}_{1}}{\partial r}\right)+\frac{{N}_{t}}{{N}_{b}r}\frac{\partial }{\partial r}\left(r\frac{\partial {T}_{1}}{\partial r}\right)-\alpha {S}_{c}{\mathrm{exp}}^{-\xi }{\beta }_{t}{T}_{1}(-\xi +n-2n\xi {\beta }_{t}{T}_{0})={L}_{e}{w}_{0}\frac{\partial {C}_{0}}{\partial z}.$$

The equation of motile microorganism is formulated as

56 $$\frac{1}{r}\frac{\partial }{\partial r}\left(r\frac{\partial {N}_{1}}{\partial r}\right)-{P}_{e}\left(({N}_{0}+\Omega )\frac{{\partial }^{2}{C}_{1}}{\partial {r}^{2}}+{N}_{1}\frac{{\partial }^{2}{C}_{0}}{\partial {r}^{2}}+\frac{\partial {N}_{0}}{\partial r}\frac{\partial {C}_{1}}{\partial r}+\frac{\partial {N}_{1}}{\partial r}\frac{\partial {C}_{0}}{\partial r}\right)={L}_{m}{w}_{0}\frac{\partial {N}_{0}}{\partial z}.$$

The suitable boundary prerequistes turn to

$${u}_{1}=0, {w}_{1}=-\frac{{\gamma }^{*}}{1+{\lambda }_{1}}(\frac{\partial {w}_{1}}{\partial r}-{\lambda }^{*}{w}_{0}\frac{{\partial }^{2}{w}_{0}}{\partial r\partial z}, \frac{\partial {T}_{1}}{\partial r}=-{\beta }_{h}{T}_{1},$$

57 $${C}_{1}={N}_{1}=0 at r={r}_{2}=r(z)+{H}_{1},$$

58 $${u}_{1}={w}_{1}={T}_{1}={C}_{1}={N}_{1}=0, \; at \; r={r}_{1}.$$

To tackle this mechanism, one can stratify the same procedure of solution that has been accomplished in the subsection “Zero-order solution in term of $\delta$”. Thence, one can carry out the following solutions:

59 $$\begin{aligned}{w}_{1}(r,z)&={a}_{68}{r}^{6}+{a}_{69}{r}^{5}+{a}_{70}{r}^{5}\mathrm{ln}r+{a}_{71}{r}^{4}+{a}_{72}{r}^{4}\mathrm{ln}r+{a}_{73}{r}^{4}{\mathrm{ln}}^{2}r+\left(\frac{(1+{\lambda }_{1}){G}_{1}(z)}{4}+{a}_{74}\right){r}^{2}\\ &\quad+{a}_{75}{r}^{2}\mathrm{ln}r+{a}_{76}{r}^{2}{\mathrm{ln}}^{2}r+({a}_{78}+{d}_{11}+{d}_{19})\mathrm{ln}r+{d}_{20}+{d}_{12},\end{aligned}$$

60 $$\begin{aligned}{T}_{1}(r,z)&={a}_{106}{r}^{10}+{a}_{107}{r}^{6}+{a}_{108}{r}^{6}\mathrm{ln}r+({a}_{47}+{a}_{109}){r}^{4}+\left(\frac{{a}_{40}}{4}+{ a}_{110}\right){r}^{4}\mathrm{ln}r+\left({a}_{48}+{a}_{111}\right){r}^{2}\\ &\quad+\left({a}_{49}+{a}_{112}\right){r}^{2}\mathrm{ln}r+\left(\frac{{a}_{42}}{4}+{a}_{113}\right){r}^{2}{\mathrm{ln}}^{2}r+{a}_{114}{\mathrm{ln}}^{2}+\left({d}_{13}+\frac{{d}_{21}-{D}_{u}{P}_{r}{d}_{15}}{1+{R}_{n}}\right)\mathrm{ln}r+{d}_{14}\\ &\quad+{d}_{22}-\frac{{D}_{u}{P}_{r}{d}_{16}}{{r}_{2}(z)(1+{R}_{n})}),\end{aligned}$$

61 $$\begin{aligned}{C}_{1}(r,z)&={a}_{125}{r}^{6}+{a}_{126}{r}^{6}\mathrm{ln}r+{a}_{127}{r}^{6}{\mathrm{ln}}^{2}r+({a}_{128}+{L}_{e}{a}_{57}){r}^{4}+({L}_{e}\frac{{a}_{53}}{16}+{a}_{129}){r}^{4}\mathrm{ln}r\\ &\quad+{a}_{130}{r}^{4}{\mathrm{ln}}^{2}r+{a}_{131}{r}^{4}{\mathrm{ln}}^{3}r+({a}_{132}+{L}_{e}{a}_{58}){r}^{2}+({a}_{133}+{L}_{e}{a}_{59}){r}^{2}\mathrm{ln}r\\ &\quad+(\frac{{L}_{e}{a}_{55}}{4}+{a}_{134}){r}^{2}{\mathrm{ln}}^{2}r+({a}_{135}+{d}_{23}+{d}_{15})\mathrm{ln}r+{d}_{24}+{d}_{16}-\frac{{N}_{t}}{{N}_{b}}{d}_{14}),\end{aligned}$$

62 $$\begin{aligned}{N}_{1}(r,z)&=({a}_{143}+{L}_{m}{a}_{57}){r}^{4}+({L}_{m}\frac{{a}_{53}}{16}+{a}_{144}){r}^{4}\mathrm{ln}r+{a}_{145}{r}^{4}{\mathrm{ln}}^{2}r+({a}_{146}+{L}_{m}{a}_{58}){r}^{2}+({a}_{147}+{L}_{m}{a}_{59}){r}^{2}\mathrm{ln}r\\ &\quad+(\frac{{L}_{m}{a}_{55}}{4}+{a}_{148}){r}^{2}{\mathrm{ln}}^{2}r{+}_{149}{r}^{2}{\mathrm{ln}}^{3}r+{a}_{150}{\mathrm{ln}}^{3}+\frac{{a}_{142}}{2}{\mathrm{ln}}^{2}r+({d}_{25}+{d}_{17})\mathrm{ln}r+{d}_{26}+{d}_{18},\end{aligned}$$

Hence, the solution of ${G}_{1}(z)$ can be designated as

63 $${G}_{1}(z)=\frac{16({\widehat{Q}}_{1}-\frac{1}{2}({R}^{2}-{r}_{1}^{2})-\frac{{\epsilon }^{2}}{4}+\frac{1}{4}({a}_{34}-2{a}_{36})({r}_{2}^{2}-{r}_{1}^{2})-\frac{{a}_{34}}{2}({r}_{2}^{2}\mathrm{ln}{r}_{2}-{r}_{1}^{2}\mathrm{ln}{r}_{1}))}{(1+{\lambda }_{1})({r}_{2}^{4}-{r}_{1}^{4})+4(2{a}_{35}-{a}_{33})({r}_{2}^{2}-{r}_{1}^{2})+8{a}_{33}({r}_{2}^{2}\mathrm{ln}{r}_{2}-{r}_{1}^{2}\mathrm{ln}{r}_{1})}$$

where the constants ${d}_{11},$ ${d}_{12},\dots .. ,{d}_{26}$ and ${a}_{33},$ ${a}_{34},\dots .., {a}_{150}$ are determined, but they are not involved in this article to reduce the length of the paper.

## Results and discussion

**Figure 2:**
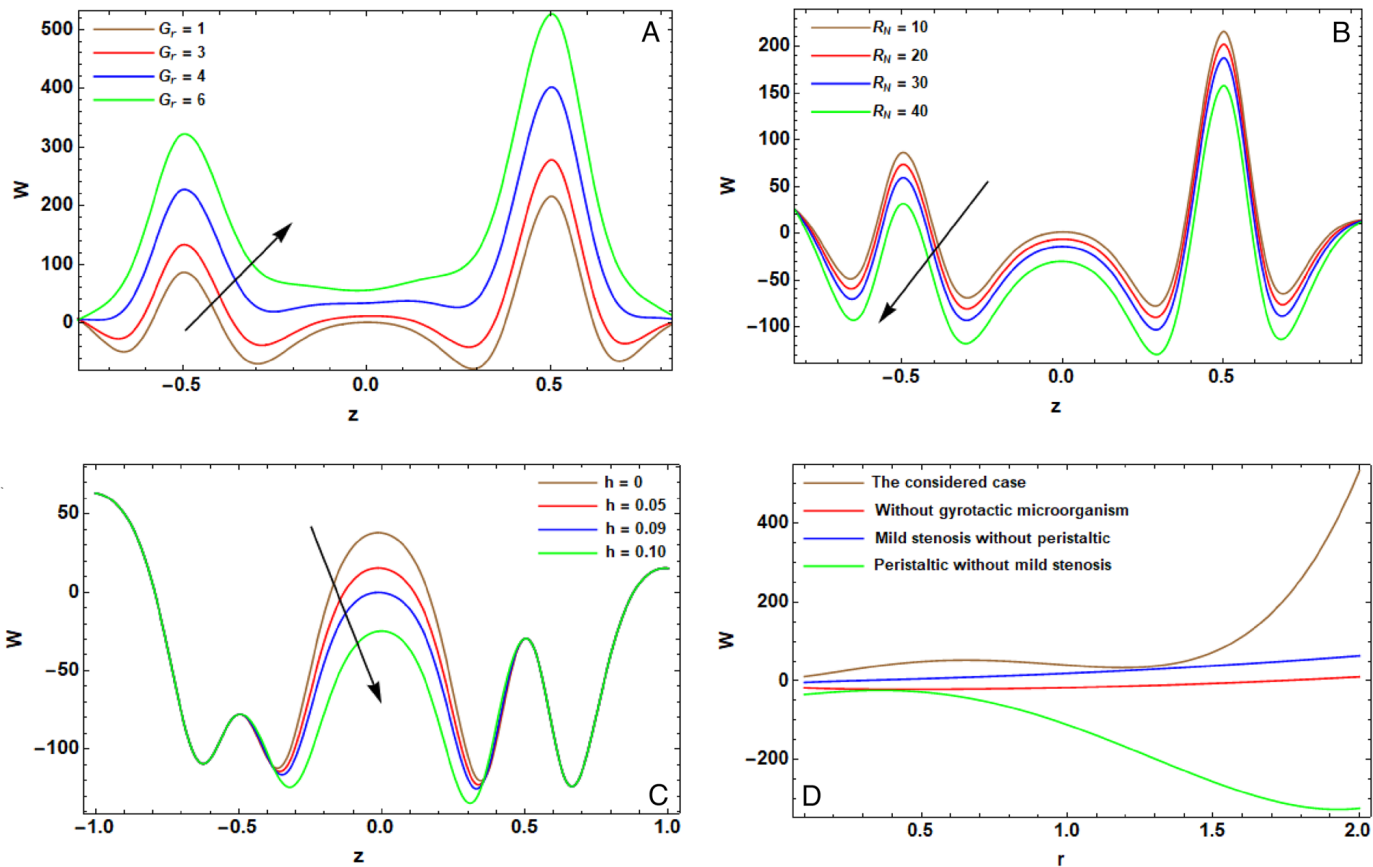
(**A**) implies alteration of $w(z)$ for assorted amounts of ${G}_{r}$. (**B**) implies alteration of $w(z)$ for assorted amounts of ${R}_{N}$. (**C**) implies alteration of $w(z)$ for assorted amounts of $h$. (**D**) implies alteration of $w(z)$ for particular status.

**Figure 3:**
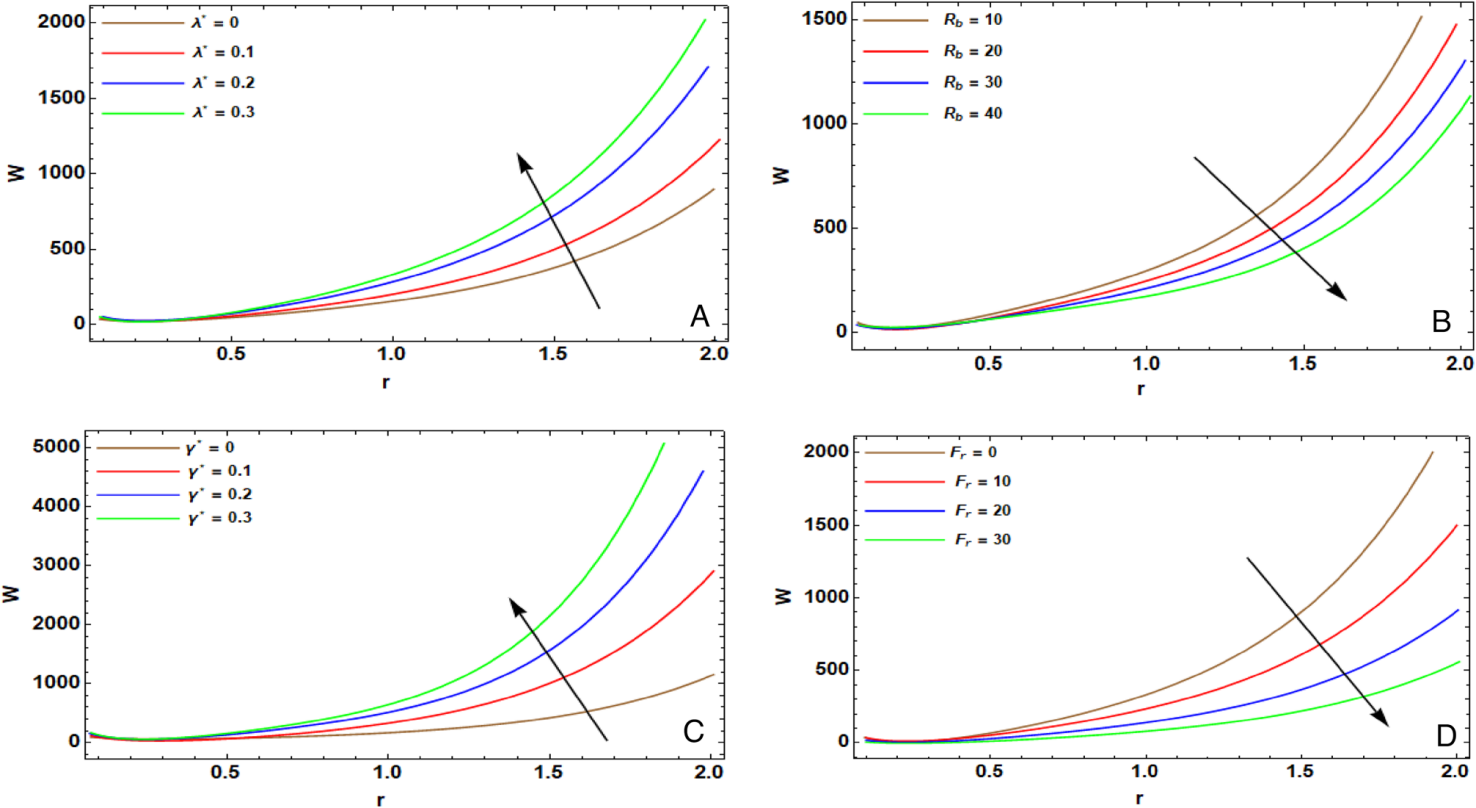
(**A**) implies alteration of $w(r)$ for various amounts of ${\lambda }^{*}$. (**B**) implies alteration of $w(r)$ for various amounts of ${R}_{b}$. (**C**) implies alteration of $w(r)$ for various amounts of ${\gamma }^{*}$. (**D**) implies alteration of $w(r)$ for various amounts of ${F}_{r}$.

**Figure 4:**
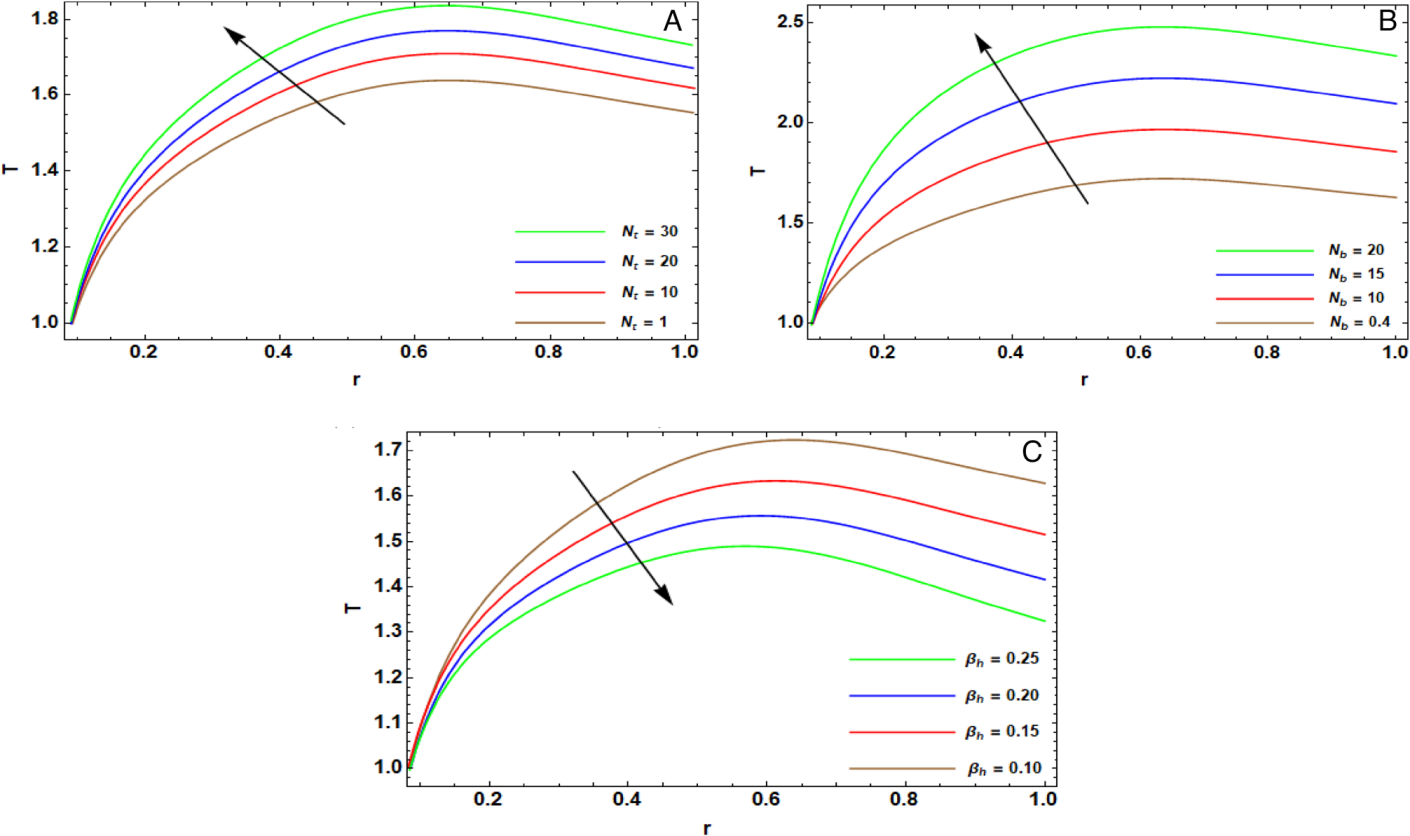
(**A**) implies alteration $T(r)$ for diverse amounts of ${N}_{t}$. (**B**) implies alteration $T(r)$ for diverse amounts of ${N}_{b}$. (**C**) implies alteration $T(r)$ for diverse amounts of ${\beta }_{h}$.

**Figure 5:**
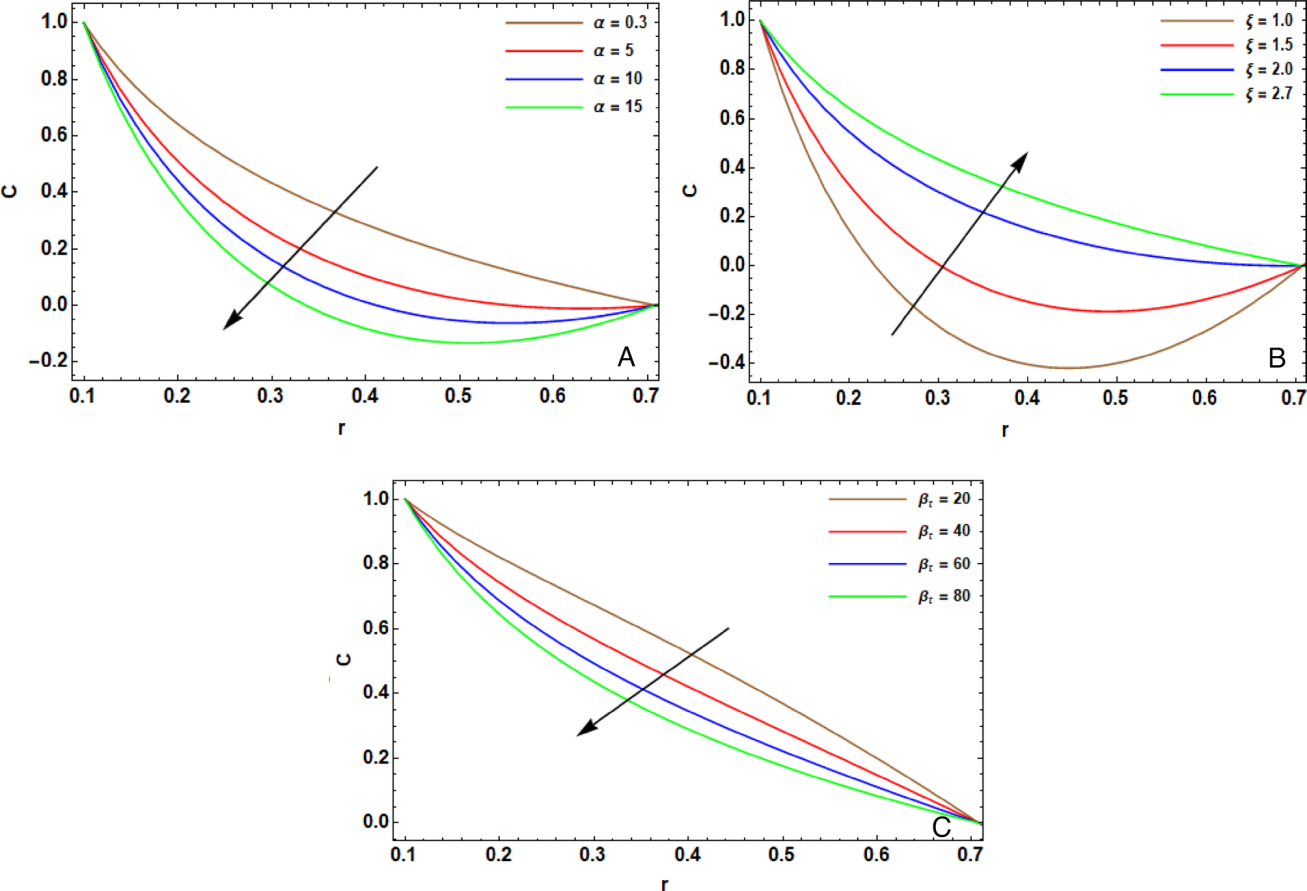
(**A**) implies alteration $C(r)$ for diverse amounts of $\alpha$. (**B**): implies alteration $C(r)$ for diverse amounts of $\xi$. (**C**) implies alteration of $C(r)$ for diverse amounts of ${\beta }_{t}$.

**Figure 6:**
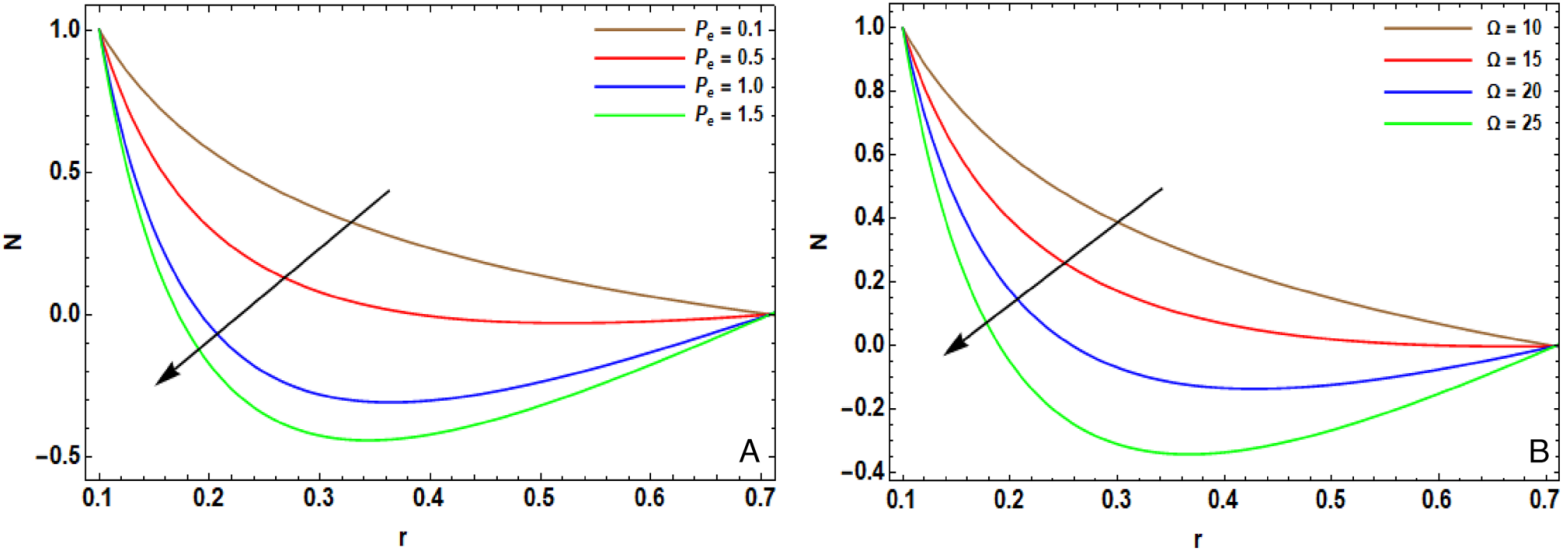
(**A**) Implies alteration $N(r)$ for diverse amounts of ${P}_{e}$. (**B**) implies alteration $N(r)$ for diverse amounts of $\Omega$.

The arithmetical program of Mathematica (version 12.0.0.0) is operated to expose the effectiveness of the diversified physical operators that are manifested in this article on the distribution of pivotal velocity $w$, temperature $T$, concentration $C$, motile microorganisms $N$ and the pressure gradient $dP/dz$. The computing range of the prevailing operators is adopted from former articles; see Refs.^10,13,16,29,35^. This range is prepared for the assorted quantities of the factors: ${H}^{2}=10$, ${r}_{1}=0.1$, ${V}_{0}^{*}=0.1$, ${\lambda }_{1}=0.9$, ${\lambda }^{*}=0.3$, ${F}_{r}=10$, ${D}_{a}=0.02$, ${G}_{r}=0.2$, ${R}_{e}=0.05$, ${R}_{N}=0.4$, ${R}_{b}=0.4$, ${N}_{t}=0.4$, ${N}_{b}=0.3$, $\alpha =0.3$, $L=2$, ${z}_{0}=0.5$, $d=2$, $m=0.2$, ${\gamma }^{*}=0.8$, $\epsilon =0.2$, $\phi =0.05$, $h=0.2$, ${R}_{n}$=0.05, $Q=0.1$, ${S}_{c}=0.3$, ${S}_{r}=0.5$, ${D}_{u}=0.1$, ${P}_{r}=0.3$, ${B}_{r}=0.3$, ${\beta }_{t}=0.01$, $\xi =0.1$, $n=0.1$, ${L}_{e}=0.2$, ${L}_{m}=0.2$, $e=0.1$, $\Omega =1.5$, ${\beta }_{h}=0.1$, ${P}_{e}=0.3$ and $\delta =0.01$. Some prime findings are diagrammatically exhibited in Figs. 2, 3, 4, 5 and 6 as follows:

### Velocity structure

The impact of local temperature Groshof numeral ${G}_{r}$ on pivotal speed $w(z)$ is exposed via Fig. 2A. ${G}_{r}$ serves a fundamental function in this investigation. Physically, in the existence of temperature transmission, ${G}_{r}$ denotes the proportion among the natural convection buoyancy force due to spatial variation in fluid’s density (caused by temperature differences) and the viscous force occurring through the fluid layers. Moreover, it governs the ratio of length scale to natural convection thickness. The buoyancy influences are fundamental via gravity and the difference of temperature. Therefore, it is detected that the increase in ${G}_{r}$ causes a boost in the velocity profile due to the enlargement of thermal buoyancy force. Moreover, ${G}_{r}>0$ stands for the heating of the nanofluid. So, the values of the axial velocity boost quickly and then decompose smoothly to the free stream velocity. This output is fully complying with the findings listed in the works of El-dabe et al.^5^.

Figure 2B substantially illustrates the effect of buoyancy in the expression of nanoparticle Groshof numeral ${R}_{N}$ on the pivotal speed $w(z)$. Nanoparticle Groshof numeral ${R}_{N}$ portrays the buoyancy force and the viscous hydrodynamic force. It is also deemed as a mensuration of the driving forces of natural concentration. So, elevation of ${R}_{N}$ increases the viscous force. Thence, the rise in ${R}_{N}$ attenuates velocity. In nature, the velocity enlarges, and the peak amount is more distinctive because of increasing in the species buoyancy force. The velocity allocation arrives a distinctive paramount value to move towards the free stream amount. Consequently, the velocity magnifies with increase of nanoparticle Groshof numeral.

The influence of the assorted quantities of maximum height $h$ on $w(z)$ is exposed in Fig. 2C. It is evident that, at ambit ($-0.5\le z\le 0.5$), the elevation of $h$ attenuates $w(z)$. Whilst, at the supplementary interval, there are no alterations in the amounts of speed. In case of ($h=0$), the amounts of velocity increase much more than those relative to the appearance of stenosis. Thus, in the state of the sickness of blood clot, the apparition of clots at the vessel attenuates blood streaming^12^. Therefore, for the diseases of blood clot, the presence of the clots, at the artery straitens the blood flow, causes a harmful impact on the body organs. This output is fully complying with the findings listed in the works of El-dabe and Mostapha^44^.

Figure 2-${\mathbb{D}}$ compares the current examination highlighted in brown curves and preceding investigations of El-dabe et al.^5^, Rahman et al. ^14^ as well as El-dabe et al.^46^, see Table 1. The previous work of El-dabe et al.^5^ examined the peristaltic transmission of a Jeffrey nanofluid throughout the tapered artery along moderate stenosis and slip stipulation in the absence of microorganisms. It is represented by the red curve, ${H}^{2}=0$, ${r}_{1}=0.1$, ${V}_{0}^{*}=0.1$, ${\lambda }_{1}=0.9$, ${\lambda }^{*}=0.3$, ${F}_{r}=0$, ${D}_{a}=0.0000001$, ${G}_{r}=0.2$, ${R}_{e}=0.05$, ${R}_{N}=0.4$, ${R}_{b}=0$, ${N}_{t}=0.4$, ${N}_{b}=0.3$, $\alpha =0$, $L=2$, ${z}_{0}=0.5$, $d=2$, $m=0$, ${\gamma }^{*}=0.8$, $\epsilon =0.2$, $\phi =0.05$, $h=0.2$, ${R}_{n}=0$, $Q=0.1$, ${S}_{c}=0.3$, ${S}_{r}=0.5$, ${D}_{u}=0$, ${P}_{r}=0.3$, ${B}_{r}=0.2$, ${\beta }_{t}=0$, $\xi =0$, $n=0$, ${L}_{e}=0$, ${L}_{m}=0$, $e=0$, $\Omega =0$, ${\beta }_{h}=0.1$, ${P}_{e}=0$ and $\delta =0.01$. Rahman et al.^14^ addressed the impacts of slip on Jeffrey nanoparticles due to tapered artery having mild stenosis in case of ignoring peristaltic transmission and microorganisms. It is expressed by the blue curve, ${r}_{1}=0.000001$, ${V}_{0}^{*}=0$, $\epsilon =0$, $Q=0$, ${S}_{c}=0$, ${S}_{r}=0$, ${P}_{r}=0$, ${B}_{r}=0$ and ${\beta }_{h}=0.1$. Nevertheless, the former work of El-dabe et al.^46^ examined the influences of MHD peristaltic movements of Jeffry nanofluid in the absence of moderate stenosis and microorganisms. This state is sketched as ${D}_{a}=0.05$, $L=0$, ${z}_{0}=0.5$, $d=2$, $m=0$, ${\gamma }^{*}=0.8$, $\epsilon =0.2$, $\phi =0$, $h=0$, $Q=0.1$, ${S}_{c}=0.3$, ${S}_{r}=0.5$ and represented in the diagram by green curves. It is revealed that the amounts of speed in our study are superior to those of previous works.

**Table 1:** Velocity field for special cases.

r	0.5	1.5	1.7
Obtained results (brown curve)	38.9	58.5	157.4
Results of El-dabe et al.^5^ (red curve)	− 0.702	− 1.2	− 1.4
Results of Rahman et al.^14^ (blue curve)	− 1.4	1	2.1
Results of El-dabe et al.^46^ (Green curve)	− 0.7	− 1.1	− 1.2

Table 1 presents a comparison between our results and open literature that mentioned before. It is clear that the results of this research state that the amounts of velocity are superior to those of previous works because of the previous assumption.

The impact of Weissenberg numeral of Jeffery type ${\lambda }^{*}$ of $w(r)$ is indicated throughout Fig. 3A. The impact of non-Newtonian fluids is achieved by considering different amounts of ${\lambda }^{*}$. It is evident that the speed enhances due to the elevation of ${\lambda }^{*}$. In the investigation, it is denoted as a dimensionless numeral of viscoelastic fluids. ${\lambda }^{*}$ is specified as the proportion among the elastic forces and the viscous forces. Therefore, the augmentation in Weissenberg numeral leads to reduction in viscous forces. Consequently, the velocity is enhanced. Further, the state of (${\lambda }^{*}$ = 0) signals to a Newtonian liquid. It is depicted that the streaming of speed curves due to a Newtonian liquid is preponderantly slighter than those in state of Jeffery type. This output is fully complying with the findings listed in the works of El-dabe et al.^5^.

Figure 3B substantially indicates the influence of biconvection Rayleigh numeral ${R}_{b}$ on $w(r)$. It is evident that ${R}_{b}$ diminishes the fluid. The motivation of diminishing the velocity of the fluid is because ${R}_{b}$ depicts the buoyancy driven influx. It can be deemed as a mensuration of driving forces of the inbred convection. So, elevation of ${R}_{b}$ leads to increase in the buoyancy force and the viscous force. Thence, the rise in ${R}_{b}$ attenuates velocity.

The influence of the slip factor ${\gamma }^{*}$ on $w(r)$ is examined throughout Fig. 3C. It is clarified that for greater slip factor, the velocity boosts. As the slip factor is denoted as the aberration in the angle at which the fluid can slither over the tube, the influence is beneficial in specifying the energy transfer between the tube and the fluid. The motivation beyond the elevation of ${\gamma }^{*}$ is that the resistance is dwindled throughout slip, which leads to boosting the velocity.

Figure 3D introduces the influence of various quantities of the Forchheimer factor ${F}_{r}$ on $w(r)$. It is observed that the growth of ${F}_{r}$ contradicts the influx and causes diminution in speed. In state of porous gap and pores bulks, elevation of ${F}_{r}$ improves the viscous force. Thence, the rise in ${F}_{r}$ promotes the flow impedance, thus the speed decelerates. Hence, the minimum velocity is related to the maximum amount of ${F}_{r}$, which illuminates that the Forchheimer quadratic drag strongly decelerates the flow rate.The finding in the case of the Darcy’s influx may be designed by ignoring ${F}_{r}$. It is revealed that the speed, in the case of the Darcy’s influx, is smaller than that in the existence of the Forchheimer term. This output is fully complying with the findings listed in the works of He and Mostapha^29^.

### Temperature structure

Figure 4A depicts the mutation of $T(r)$ for various amounts of thermophoresis factor ${N}_{t}$. It is evident that the temperature enlarges with the enhancement of the thermophoresis factor. Physically, the thermophoresis circumstance is concerned with the transport of nanoparticles from a hot district to a cold district out of the temperature difference. Furthermore, it determines the impact of thermophoretic force on the nanofluid temperature and nanoparticle volume fraction. Therefore, the enelarging in the amount of ${N}_{t}$ causes an increase in the alteration among the wall and temperatures. Moreover, the thermophoretic force can conflict with the motion, and consequentally, the temperature alteration is boosted. This output is fully complying with the findings listed in the works of Rahman et al.^14^.

The influence of the assorted values of the Brownian motion factor ${N}_{r}$ on $T(r)$ is exposed in Fig. 4B. It is depicted that the expansion in the amounts of the Brownian factor magnifies the temperature. In the vision of physical concern, Brownian motion is denotes as the random motion of particles suspended in a fluid that causwed by their collision with the quick atoms or molecules in the fluid. The Brownian factor is concerned with the desultory movement of particles on the surface, and the elevation of ${N}_{r}$ ameliorates this movement of the fluid particles, which results in revolving and oscillations of molecules with the kinetic energy and produces more heat. This output is fully complying with the findings listed in the works of El-dabe et al.^5^.

Figure 4C depicts the effect of the assorted quantities of Biot numeral ${\beta }_{h}$ on $T(r)$. The Biot numeral is the proportion of the thermal resistance for conduction through an object to the resistance for convection at the surface of this object. This proportion denotes to the temperature inside an object when the object is heated or cooled over time by a heat flux. It is realized that temperature deflates with the elevation of ${\upbeta }_{\mathrm{h}}$. In deed, thermic conductivity dwindles with the augmentation of ${\beta }_{h}$, which results in reducing the temperature profile. This output is fully complying with the findings of the work of El-dabe and Mostapha^26^.

### Concentration of nanoparticles

Figure 5A represents the diversity of concentration $C(r)$ for distinct quantities of the chemical reaction factor $\alpha$. Physically, the chemical interaction is recognized as a dimensionless numeral which stands for the proportion of thermal diffusivity to mass diffusivity. It cancharacterize flows with heat and transfer. Therefore, it improves the interfacial mass transition average which gives rise in the intermolecular force. Thence, the concentration structure contracts. This impact clears that the mass transfer average boosts through a high destructive chemical rate, causing a decline in concentration and gyrotactic microorganism. This essential result is in a good agreement with this obtained by Majeed et al.^37^.

Figure 5B describes the influence of different values of the activation energy factor $\xi$ on $C(r)$. It is indicated that there is rush onwards in activation energy which causes augmentation in the concentration structure. The Arrhenius expression is used to describe the mathematical formula of activation energy. In deed, it is revealed that the drop in heat and acceleration are attributed to a weak reaction average constant. In this procedure, this reduces chemical interaction and induces higher concentration. This motivation is tackled to transit drug to the site of the malign tissue to recuperation. This essential result is in a good agreement with this obtained by Ellahi et al.^16^.

The structure of concentration $C(r)$ for distinct amounts of the temperature ratio factor ${\beta }_{t}$ is scrutinized in Fig. 5C. The temperature ratio factor yeilds a noteworthy drop in concentration. As the variance among the temperature of the fluid and the wall temperature broadens, the thickness of the concentration boundary layer is widened. This thickness hampers the elevation of concentration.

### Motile microorganism density

The alteration of motile microorganism density $N(r)$ for distinct amounts of bioconvection Peclet numeral ${P}_{e}$ is outlined in Fig. 6A. It is recognized as the proportion of the average of advection of a physical object to the average of diffusion of this object. Physically, the Peclet numeral is the product of the Reynolds numeral and the Schmidt one (Re × Sc). It is also defined as product of the Reynolds numeral and the Prandtl numeral (Re × Pr). A flow will often have different Peclet numeral for heat and mass. This can lead to the phenomenon of double diffusive convection. It is detected that the elevation in ${\mathrm{P}}_{\mathrm{e}}$ decreases the motile microorganism density, since robust Peclet numeral denotes weaker Brownian coefficients which cause little penetration of swimming microorganisms. Further, in the existance of bioconvection Peclet number, the rate swimming velocity of the motile bacteria boosts concerned with the base fluid. Hence, the density of microorganisms declinds. This output is fully complying with the findings of the work of Majeed et al.^36^.

Figure 6B interprets the influence of the bioconvection constant $\Omega$ on $N(r)$. The motile microorganism density is reduced by the elevation of bioconvection constant. In deed, the impacts of Lorentz force on density fluid as well as on shear stress are more intrinsic.

### Pressure gradient features

**Figure 7:**
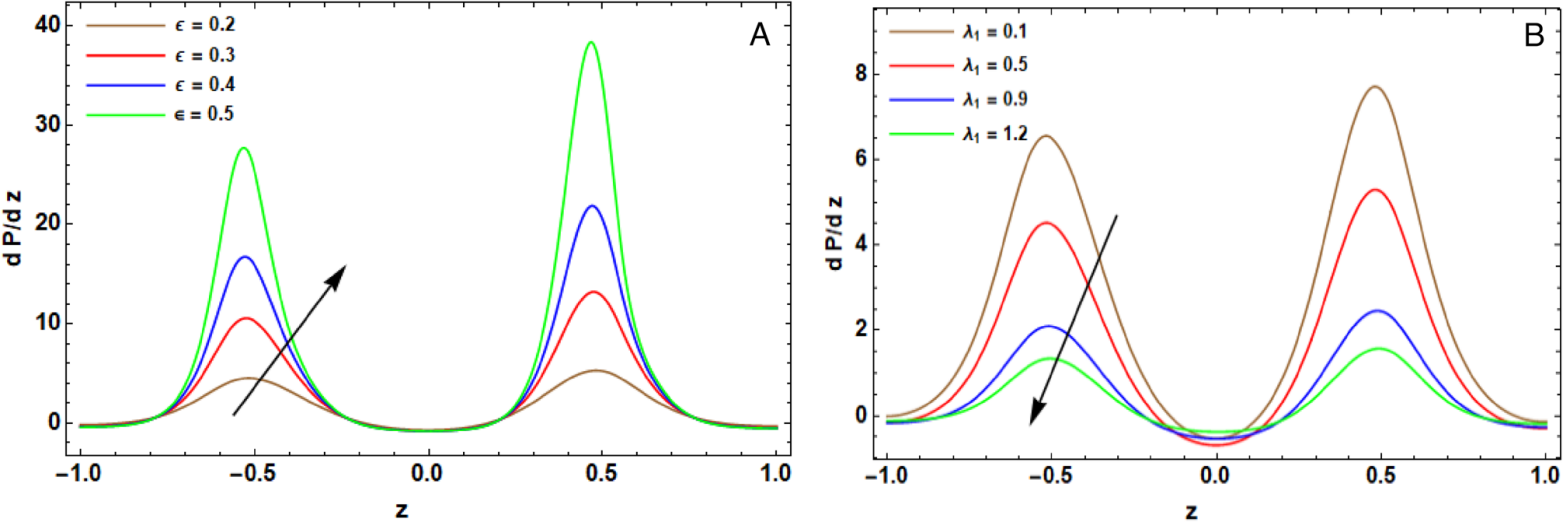
(**A**) implies alteration of $\frac{dP}{dz}$ for diverse amounts of $\epsilon$. (**B**) implies alteration of $\frac{dP}{dz}$ for diverse amounts of ${\lambda }_{1}$.

The influences of the assorted quantities of the amplitude ratio $\epsilon$ and the ratio of relaxation to retardation time ${\lambda }_{1}$ on the change of the pressure gradient $\frac{dP}{dz}$ with $z$-axis are clarified throughout Fig. 7A and B, respectively. It is evident from these diagrams that the curves brings about a periodic style in the $z$-axis. There are also two varied peaks relating to the maximum pressure gradient at $z=-\mathrm{0.5,0.5}$ and the minimum pressure gradient happening at $z=-\mathrm{1,0},1$. These peaks protrude throughout the sinusoidal shape of the frontier.

From Fig. 7A, the pressure gradient exhibits an amelioration modality with improvements in the amplitude ratio $\epsilon$ due to the utmost rise tacking place at $z=-\mathrm{0.5,0.5},$ which deals with the wave compression. Further, there is an interval in the surrounding district of the wave, where $\frac{dP}{dz}$ exposes a less trend with the elevation of $\epsilon$.

Figure 7B detects that $\frac{\partial P}{\partial z}$ has a detraction manner with boosting in the ratio of relaxation to retardation time happening at $z=-\mathrm{0.5,0.5}$ , which deals with the wave compression. The augmentation in ${\lambda }_{1}$ causes detraction in the viscosity of fluid, which leads to diminishing in the pressure gradient.

## Conclusion scheme

In this work, an unsteady locomotion of a Jeffery nanofluid comprising motile gyrotactic microorganisms passing through endoscope is exposed. There locates a sinusoidal wave transit over the wall of the outer anisotropically stenosed tube. The influx proceed down via a Darcy-Forchheimer porous surrounding. A stable sturdy magnetic field has acted upon the framework. Hall and Joule heating actions are considered. The current paper investigates thermic radiation as well as response of chemical interaction with Soret and Dufour scheme. Activation energy has been implemented to the concentration of nano-particles due to the amended Arrhenius scheme and the Buongiorno type. Further, the slip stipulation is deemed relative to the speed scheme. Meanwhile, Convective stipulation is implemented for temperature. The proposition of protracted wavelength besides subdued Reynolds numeral is regulated to transit the manner of partial differential formulations which transform this phenomenon to ordinary one. Homotopy perturbation manner is tackled to manage the traditional solutions of generated neutralizations. The analytical solutions are exhibited in pictorial formula for speed, nanoparticle temperature, nanoparticle concentration, motile microorganisms, and pressure gradient.

The conclusion may be summed as:

1. An augmenting in local temperature Groshof numeral ${G}_{r}$ causes a boost in the velocity profile. Nevertheless, Nanoparticle Groshof numeral ${R}_{N}$ portrays the buoyancy force and the viscous force. So, elevation of ${R}_{N}$ increases the viscous force. Thence, the rise in ${R}_{N}$ attenuates velocity.
2. The speed improves due to the elevation of Weissenberg numeral of Jeffery type ${\lambda }^{*}$, as the augmentation in Weissenberg numeral leads to reduction in viscous forces.
3. The temperature rises with the enhancement of both the Thermophoresis factor ${N}_{t}$ and the Brownian motion factor ${N}_{r}$.
4. There is growth in activation energy which causes augmentation in the concentration structure. The Arrhenius expression is used to describe the mathematical formula of activation energy. In deed, it is revealed that the drop in heat and acceleration are attributed to a weak reaction average constant.
5. The temperature ratio factor brings about a noteworthy drop in concentration. As the variance between the temperature of the fluid and the wall temperature broadens, the thickness of concentration boundary layer is widened.
6. An elevation in Peclet numeral ${P}_{e}$ decreases the motile microorganism density, since robust Peclet numeral implies to stands for weaker Brownian coefficient which causes little penetration of swimming microorganism.
7. The pressure gradient exhibits an amelioration modality with improvements in the amplitude ratio $\epsilon$. Further, it drops with boosting in the ratio of relaxation to retardation time.

Factually, the implementations of nanotechnology in the medical field has drawn considerable attention through the efficient function of chemical interaction as well as activation energy, since nanoparticles facilitate tackling varies ailments by the help of the peristaltic transition of blood to convey drugs to the deteriorated tissue. The current study offers a medication for the malign cells and clogged arteries of the heart by penetrating a slender tube (catheter). Also, the study at hand addresses the gastric juice movement in small intestine when an endoscope is permeating across it.

## Data Availability

All data generated or analyzed during this study are included in this manuscript.

## List of symbols

$\underline{\underline{A}}$: Rate of strain tensor
$${a}_{0}$$: Wave amplitude
$\underline{B}$: The external magnetic field
$${B}_{r}$$: Brickmann numeral
$b$: The chemotaxis constant of the microorganism
$$C$$: Nanoparticle concentration
${c}_{b}$: The Forchheimer factor
${C}_{e}$: Coefficient of nanoparticle expansion
${C}_{s}$: The concentration susceptibility
$D$: Mass diffusivity factor
$${D}_{a}$$: Darcy factor
${D}_{B}$: Brownian diffusion factor
${D}_{m}$: The diffusion factor of the microorganisms
${D}_{T}$: The thermophoretic diffusion factor of nanoparticle
$${D}_{u}$$: Dufour number
$${E}_{a}$$: Activation energy
$${E}_{c}$$: Eckert numeral
$e$: The electron charge
$$\frac{1}{e{n}_{c}}$$: Hall factor
$${F}_{r}$$: Forchheimer numeral
${G}_{r}$: Thermal Rayleigh numeral or the local temperature Grashof numeral
$\underline{g}$: The gravitational force
$${H}^{2}$$: Hartmann numeral
${H}_{0}$: The height of stenosis
$h$: The maximum height of stenosis
${h}_{t}$: The heat transfer factor
$\underline{J}$: The current density
$K$: The thermic conductivity
${K}_{0}$: Rosseland absorption coefficient
${K}_{r}$: Average of reaction
${K}_{T}$: Thermic diffusion ratio
$k$: The thermometric conductivity
${k}_{1}$: The permeability factor of porous moderate
$$\frac{kt}{{R}_{1}}$$: Wave speed
${L}_{e}$: Nanoparticles Lewis numeral
${L}_{m}$: Microorganism Lewis number
$m$: Hall current factor
${m}_{1}$: Slope of anisotropically outer tube
$N$: Density of motile microorganism
${N}_{b}$: Brownian motion factor
$${N}_{t}$$: Thermophoresis factor
$n$: The fitted rate
${n}_{c}$: Density of free electrons
$$P$$: The pressure
$${P}_{e}$$: Peclet numeral
$${P}_{r}$$: Prandtl numeral
${q}_{r}$: The radiative temperature influx
$R(z)$: The radius of nonuniform outer tube
${R}_{b}$: Biconvection Rayleigh numeral
$${R}_{e}$$: Reynold’s numeral
${R}_{N}$: Nanoparticles Grashof numeral
$${R}_{n}$$: Radiation factor
$\underline{\underline{S}}$: The stress deviator
$${S}_{c}$$: Schmidt numeral
$$T$$: The temperature
${T}_{e}$: Base fluid volumetric coefficient of thermic expansion
$\underline{V}$: Speed of the fluid
${z}_{0}$: The half-length of stenosis
$$\delta$$: The wavelength
$\phi$: The angle of anisotropically tube
$$\mu$$: Dynamic viscosity
$$\lambda$$: Wave length
${\lambda }^{*}$: Weissenberg numeral of Jeffery type
${\lambda }_{1}$: Proportion of relaxation time to retardation one
${\lambda }_{2}$: The retardation time
${\rho }_{f}$: The effective fluid density
${\rho }_{m}$: Density of microorganisms
${\rho }_{p}$: Density of the nanoparticles
$(\rho c{)}_{f}$: The heat capacity of fluid
$(\rho c{)}_{p}$: Effective heat capacity of nanoparticle
$\sigma$: The electric conductivity
${\sigma }_{0}$: Stefan Boltzman factor
$\vartheta$: Average volume of microorganisms
$$\omega$$: Boltzmann constant
${\omega }_{e}$: The maximum cell of swimming speed
$$\nu$$: Kinematic viscosity
$$\epsilon$$: Amplitude proportion
$\alpha$: Reaction rate constant
$\beta$: Adverse heat gradient
${\beta }_{t}$: Temperature proportion factor
${\beta }_{h}$: Biot numeral of heat transfer
$$\gamma$$: Slip factor
${\gamma }^{*}$: Non-dimensional slip factor
${\tau }_{c}$: Proportion among effective heat capacity of the nonoparticles material and capacity of fluid
$\xi$: Non-dimentional activation energy factor
$$\Omega$$: Bioconvection constant
